# High- or Intermediate-Risk Histologic Features in Patients with Clinical Early-Stage Cervical Cancer Planned for Fertility-Sparing Surgery: A Systematic Review

**DOI:** 10.3390/cancers15153920

**Published:** 2023-08-01

**Authors:** Janneke T. Wolswinkel, Mieke L. G. ten Eikelder, Cornelia G. Verhoef, Petra L. M. Zusterzeel

**Affiliations:** 1 Department of Obstetrics and Gynecology, Radboud University Medical Center, P.O. Box 9101, 6500 HB Nijmegen, The Netherlands; janneke.wolswinkel@radboudumc.nl (J.T.W.); mieke.teneikelder@radboudumc.nl (M.L.G.t.E.); 2 Department of Radiation Oncology, Radboud University Medical Center, P.O. Box 9101, 6500 HB Nijmegen, The Netherlands; lia.verhoef@radboudumc.nl

**Keywords:** fertility-sparing surgery, cervical cancer, lymph node metastases, parametrial involvement, positive surgical margin, intermediate risk, high risk

## Abstract

**Simple Summary:**

Fertility-sparing surgery (FSS) is a viable option for young women with early-stage cervical cancer, with the success of preserving fertility rate exceeding 90%. However, certain high-risk histologic features such as positive lymph nodes, positive surgical margins, parametrial invasion or intermediate-risk factors may require additional treatments that can negatively affect fertility. This review provides an overview of the occurrence of these high- or intermediate-risk histologic features, the available treatment options and the variations in approaches among different treatment centers worldwide. Parametrial invasion in tumors less than 2 cm was found to be rare, supporting the rationale for omitting parametrectomy in small tumors. In cases of positive lymph nodes at frozen section analysis, a hysterectomy is not recommended prior to adjuvant (chemo)radiation, as leaving the uterus in place reduces the risk of complications during surgery and radiation therapy. Adjuvant chemotherapy after FSS could be an alternative treatment option considering its effectiveness, safety and potential for fertility preservation.

**Abstract:**

Background: Fertility-sparing surgery (FSS) is a viable option for young women with early-stage cervical cancer (ECC); however, certain risk factors may necessitate additional treatments and impact fertility. This review examines the prevalence of these risk factors and available treatment options. Methods: A systematic search was conducted of studies including patients with ECC (IA1 with LVSI, IA2, IB1 (FIGO 2009)) who underwent FSS. Results: Sixty-four articles, comprising a total of 4118 women planned for FSS, were included. High- or intermediate-risk histologic features were found in 638 (15.5%) women: 5.1% had positive lymph node(s), 4.1% had positive resection margins, 0.3% had parametrial involvement, 1.0% had unspecified high-risk features, and 5.1% had intermediate-risk histology (primarily based on the Sedlis criteria). Adjuvant treatment impaired fertility in all women with adjuvant hysterectomy and/or (chemo)radiation (58.7%). Adjuvant chemotherapy was given to 1351 (32.8%) patients, which may reduce fertility. Conclusions: Fertility preservation could be achieved in most women; but high- or intermediate-risk factors necessitate more extensive surgery or radiotherapy leading to infertility. Adjuvant chemotherapy could be an alternative treatment option considering its effectiveness, safety and higher change in fertility preservation. The low incidence of parametrial involvement justifies waiving parametrectomy in tumors < 2 cm.

## 1. Introduction

Early-stage cervical cancer (ECC) often occurs in young women that have not yet completed their reproductive phase of life. On a yearly basis worldwide, over 178,000 women younger than 45 years are diagnosed with cervical cancer [1]. Cervical cancer (CC) in a clinical early stage is traditionally treated with a (radical) hysterectomy with lymphadenectomy and in a more advanced stage with chemoradiation, which all result in the loss of child-bearing ability. Fertility-sparing surgery (FSS) is nowadays offered to young women with clinical ECC with child wish. It has been proven to be a safe alternative if performed in carefully selected patients [2]. FSS in women with clinical stage IA1 with LVSI, IA2 and IB1 (FIGO 2009) cervical cancer may involve a large loop excision of the transformation zone (LLETZ), conization or (radical) trachelectomy combined with lymph node assessment [3,4]. A (radical) trachelectomy can be performed vaginally, abdominally or with minimally invasive surgery (MIS) (laparoscopy- or robot-assisted). Pregnancy rates after these procedures differ: 56.3% after simple trachelectomy, 58.7% after radical vaginal trachelectomy and 36% after abdominal radical trachelectomy [5]. 

Women with unexpected histologic features may need adjuvant treatment that can impair their fertility. These unexpected histologic features can be categorized as high-risk, involving either positive lymph nodes, parametrial invasion or positive resection margins (Peter’s criteria) [6], or can be categorized as intermediate-risk, in accordance with Sedlis et al.: (1) LVSI plus deep one-third cervical stromal invasion and tumor of any size; (2) the presence of LVSI plus middle one-third stromal invasion and tumor size > 2 cm; (3) the presence of LVSI plus superficial one-third stromal invasion and tumor size > 5 cm; and (4) no LVSI but deep or middle one-third stromal invasion and tumor size > 4 cm [7,8]. The Sedlis criteria consist of a risk-based algorithm to discover patients with a recurrence risk of >30%, in whom radiotherapy can achieve a 47% risk reduction [8]. These criteria are currently under debate, as Levinson et al. developed histology-specific nomograms predicting a more accurate risk of recurrence [9].

Careful selection of suitable candidates for FSS is thus paramount, as the utilization of multiple treatment modalities in the event of requiring adjuvant treatment can elevate morbidity rates and reduce fertility options. Tumor size and parametrial invasion can be assessed by clinical examination with or without anesthesia and magnetic resonance imaging (MRI) [10]. To assess lymph node metastasis, computed tomography (CT), MRI or positron emission tomography (PET) CT are used. Despite careful preoperative screening and selection of patients, it cannot be avoided completely that suspicious lymph nodes or the parametrium are discovered during surgery. Then, frozen section analysis (FSA) can provide a solution. The results of FSA could lead to a change in treatment plan, mostly canceling FSS, and has a sensitivity of 72.4% for macro-metastases but only 9.5% sensitivity for micro-metastases [11]. In the majority of cases, high- and intermediate-risk features are not discovered until final histology.

Adjuvant treatment options offered worldwide vary significantly, with most treatments impairing fertility (e.g., radiation or hysterectomy) while others (such as chemotherapy) still offer a reasonable chance of conception and childbirth. This article is a systematic review on high- and intermediate-risk features in ECC patients planned for FSS. Our aim is to provide an overview about the occurrence of high- and intermediate-risk features, as well as treatment options in the case of adverse histology during or after surgery.

## 2. Materials and Methods

This article is a systematic review of the current literature on FSS for ECC and the high- and intermediate-risk features. The design of this study and result reporting was performed based on recommendations from the Preferred Reporting Items for Systematic Reviews and Meta-Analyses (PRISMA) statement [12].

### 2.1. Search Method

We conducted a systematic search of the PubMed, Embase, Cochrane Library and Web of Science databases on 27 August 2022. The search strategy included synonyms for “cervical cancer” and “fertility-sparing surgery” and “lymph nodes” (Supplementary Materials S1). EndNote library was used to remove duplicates from search results. Studies performed after 2000 in Dutch, English or French language were then selected. 

### 2.2. Study Selection

The articles were screened in EndNote for relevance on title/abstract and full text by J.W. and M.E. independently. In case of discrepancies, P.Z. was consulted. Inclusion criteria were studies that included patients with ECC (IA1 LVSI pos, IA2, IB1 (FIGO 2009)) that underwent FSS with sentinel node (SN) and or lymphadenectomy. Exclusion criteria were studies that included patients with neo-adjuvant chemotherapy (NACT), cervical cancer in pregnancy or pediatric tumors, treatment for premalignancies and systematic reviews. (Conference) abstracts were included when enough information was provided answering the main research question. Case reports and case series were excluded; cohort studies were included when the cohort consisted of at least 10 patients. Studies that prescribed NACT in a minority of patients were included in the review as long as no high- and intermediate-risk features occurred in the NACT patients. When this was not clearly described, we contacted the authors for additional information. Authors of two studies provided us with extra information on this subject [13,14]. Studies were included in the review when they at least reported on the occurrence of high- and intermediate-risk features. To prevent data set inflation, articles were compared on study groups, countries, cities and hospitals. When a suggestion of duplicate patient information existed, only the largest and most detailed patient series were included. 

### 2.3. Data Extraction

J.W. and M.E. extracted the data into an electronic database. The following variables were recorded: first author, country of origin, year of publication, years of inclusion, cohort size, clinical FIGO stage and FIGO classification used (2009 or 2018), type of work-up (MRI, PET, chest X-ray), age, histology, depth of invasion, presence of lymph vascular space invasion (LVSI), tumor size, type of intervention (LLETZ, conization, simple or radical trachelectomy, vaginal, abdominal or with MIS), type of high- and intermediate-risk features (positive lymph node (ITC, micro-metastasis, macro-metastasis)), positive margin (and used cut-off), parametrial invasion, intermediate-risk histology (criteria used), frozen section analysis or final histology, treatment after high- and intermediate-risk features (conversions to hysterectomy, adjuvant treatment (chemotherapy, (chemo)radiation therapy), ovariopexie or other fertility-sparing treatment), preservation of fertility, months of follow-up, recurrences, median time to recurrence, deaths from disease, disease-free survival (DFS) and overall survival (OS), pregnancies after trachelectomy and pregnancy outcomes. 

In a few patients, there was a combination of high-risk histology results, e.g., positive lymph node and positive surgical margin. These patients were counted in both subgroups for the subgroup analysis but only once in the total group. 

Fertility preservation was defined as patients that were not treated with either hysterectomy or radiotherapy; in patients that received only chemotherapy, fertility was considered to be feasible. 

### 2.4. Quality Assessment

Quality assessment was performed using the Risk Of Bias In Non-randomized Studies of Interventions (ROBINS-I) tool [15]. Studies were assessed on risk of bias (confounding, classification of interventions, deviation from intended intervention, missing data, selection of patients) and time-varying confounding. Although the number of variables recorded in the different studies greatly varied, no studies were excluded based on the quality score.

### 2.5. Data Synthesis

Descriptive statistics were used to analyze the frequencies of high- and intermediate-risk features and subsequent treatments. 

## 3. Results

**Figure 1:**
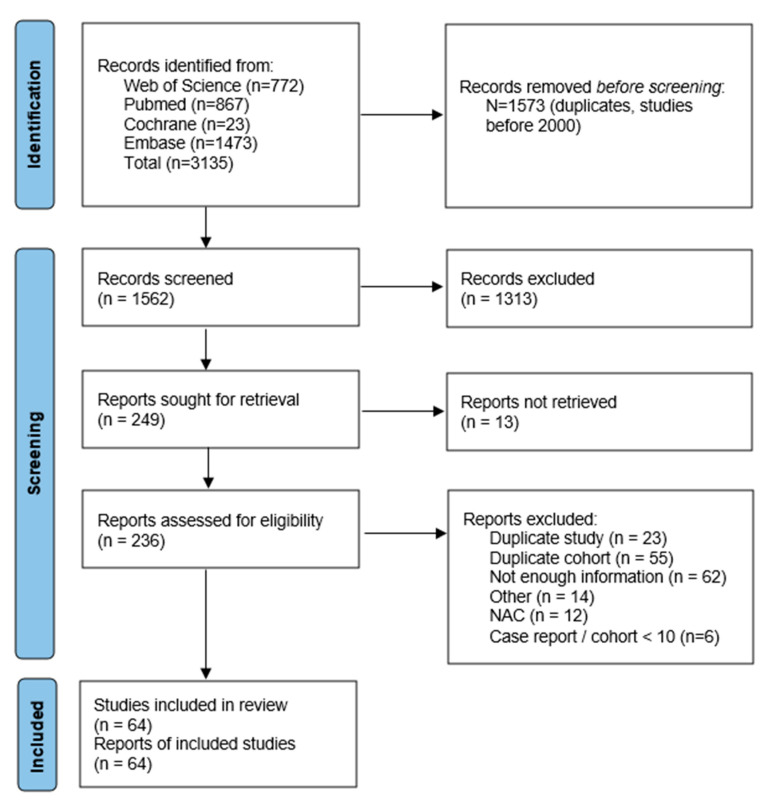
Search results.

In total, 3135 articles were collected from the electronic database search: 1473 from Embase, 867 from PubMed, 772 from the Web of Science and 23 from the Cochrane Library. The PRISMA process of article selection was followed. During the full-text review, articles were mainly excluded because of duplicate cohorts. Secondly, articles were excluded because only superficial information was provided, e.g., “wider spread of disease” or “extra tissue removed”. Sixty-four articles were included in this review. They were published between 2003 and 2022, the majority (65,6%) in the last 10 years. The overview of articles included and excluded from this review is listed in Figure 1. The risk of bias varied among different studies (Supplementary Material Table S1).

In the 64 articles, a total of 4118 women were identified who were planned for a fertility-sparing surgical procedure; the overview is shown in Table 1. A total of 66.4% of these women (2736 patients) had a tumor size < 2 cm, 12.2% (502 patients) had a tumor size > 2 cm, and for 21.4% (880 patients), the tumor size was unknown. 

In total, 638 patients (15.5%) had high- or intermediate-risk histology. Of the 431 (10.5%) patients with high-risk histology, 5.1% had positive lymph node(s), 4.1% had positive resection margins, and 0.3% showed parametrial involvement. Two patients had more than one high-risk factor. In 39 women (1.0%), a high-risk factor was found but not further specified. High-risk factors were discovered during surgery with FSA in 45.0% and in 55.0% on final histology. Intermediate risk was recognized in all except two cases in the final histology and found in 209 women (5.1%). These results are shown in Table 2.

Most women planned for FSS preserved their fertility (*n* = 3744, (90.9%)). In 9.1% (374 women) of the total population, fertility was not preserved due to more extensive surgery than originally planned (hysterectomy (181 patients, 4.4%) or adjuvant radiotherapy (*n* = 79, 1.9%)). In 2.8%, it was clear that fertility was not preserved, without any more details provided (Table 2). 

In most women with high- or intermediate-risk histology, an RVT (272 women, 42.6%) or ART (199 women, 31.2%) was performed (Table 3). 

### 3.1. Lymph Node Metastases

Positive lymph nodes were found in 212 patients (5.1%). These nodes were found during surgery with FSA in 49.5% and on final histology in 44.8%. In 5.7%, it was unknown if the positive node was diagnosed during or after surgery. Eight patients (3.7%) with a positive lymph node only had isolated tumor cells (ITC); none of them received adjuvant treatment. 

Of the 105 patients diagnosed with positive lymph nodes during surgery, 47 received extended surgical treatment: a hysterectomy (*n* = 35), hysterectomy and para-aortal lymph.

In 9.5% (10 of the 105 patients), the surgical procedure was discontinued; they received either radiotherapy or chemoradiation. Of the 95 patients diagnosed with positive lymph nodes on final histology, 12 received adjuvant surgical treatment with or without adjuvant chemotherapy or (chemo)radiation. In the 83 patients who did not undergo extra surgery, treatment was either (chemo)radiotherapy (*n* = 46), chemotherapy (*n* = 16) or unknown treatment (*n* = 7). Fourteen patients did not receive any adjuvant treatment, two of whom refused advised treatment. 

For 12 patients, it was unclear whether they were diagnosed during the FSA of final histology (Table 2). 

Forty-four studies described patients with lymph node metastasis; in fourteen of these studies, the type of lymph node metastasis (macro-metastasis, micro-metastasis, isolated tumor cells) was described. From these studies, four patients with macro-metastasis were treated with (chemo)radiation or chemotherapy with or without radical surgery. A total of 25 patients with micro-metastasis were treated with common iliac and para-aortic lymph node dissection (2 patients), (chemo)radiation (6 patients), hysterectomy with or without adjuvant treatment (4 patients), no treatment (11 patients) or unknown treatment (2 patients). The eight patients with ITC were not treated.

In 14.6% of patients with positive lymph nodes (31 patients, 0.8% of the total population), fertility was preserved, and 7.5% of patients with positive lymph nodes received chemotherapy. 

### 3.2. Positive Surgical Margins

Surgical margins were positive in 169 patients (4.1%): 166 patients had a positive surgical margin of the remaining cervix, 2 patients had a positive vaginal margin, and 1 patient was diagnosed with vesicouterine involvement. In 15 (39.5%) articles, a clear cut-off margin was described. The majority of articles (73.3%) used a cut-off of 5 mm. For the others, there was a large variation between 2 and 10 mm. 

More than half of the positive margins (52.1%) were found on FSA, and 30.8% of positive resection margins were diagnosed at final histology. In 29 patients (17.2%), this information was not provided. Whether or not an extra strip of cervical tissue was removed was not clearly documented in most studies. The majority of patients with positive uterine margins underwent a hysterectomy (*n* = 98; 58.0%). A total of 8 patients (4.7%) received (chemo)radiation therapy, 1.8% received chemotherapy, and 38 patients (0.9%) received unknown extra treatment. Fertility remained in 13.0% of patients with positive margins because a second cone or LLETZ was performed, because adjuvant treatment was refused (*n* = 1) or because they received adjuvant chemotherapy (*n* = 3) (Table 2). 

### 3.3. Parametrial Involvement

Parametrial involvement was found in just 11 patients (0.3% from the total group): 1 patient had a tumor >2 cm, 6 patients had tumors <2 cm, and in 4 patients, the tumor size was unknown. In one patient (9.1%), parametrial involvement was diagnosed at FSA, and a radical hysterectomy was performed subsequently. In the other 10 (90.9%) patients, parametrial involvement was diagnosed after final histology, and 8 of them received (chemo)radiation. In two patients, this information was not provided. Details of the (chemo)radiation treatment were not available (Table 2). In none of the patients, fertility was preserved. 

### 3.4. Intermediate-Risk Factors

In 5.1% of patients (209 patients), intermediate-risk criteria were present. In only one of these patients, intermediate risk was identified with the strict use of the Sedlis criteria. In all other cases, unknown or adapted criteria from Sedlis were used. The criteria were as follows: poor differentiation, deep stromal invasion, LVSI and tumor size >2 or >4 cm. In most articles, a combination of criteria was used (Supplementary Table S2). In all but two patients, this was diagnosed after final histology. The majority received adjuvant chemotherapy (87.1%); other modalities were a hysterectomy or adjuvant radiotherapy. A total of 1.9% of patients (four patients) refused adjuvant treatment. Fertility could be preserved in 89,0% of patients with intermediate-risk factors. 

### 3.5. Oncological and Fertility Outcome

For most patients, information about follow-up and recurrence was lacking, which makes it not feasible to analyze this data. Pregnancy rates and outcomes were not reported separately for the patients with high- or intermediate-risk factors. 

## 4. Discussion

This systematic review focused on fertility-sparing surgery for presumed early-stage cervical cancer and identified the absence or presence of high- or intermediate-risk histologic tumor characteristics in women undergoing FSS. We found that 638 (15.5%) of all women had either high-risk factors (lymph node metastases, parametrial invasion, positive resection margins) or intermediate-risk factors, necessitating an adaptation in their treatment. In 374 (9.1%) of all women, fertility could not be preserved as they underwent a hysterectomy or adjuvant (chemo)radiation. However, in 264 (6.4%) of women with one of these features, fertility was likely spared, mostly because they only received chemotherapy. 

A total of 212 (5.1%) of the patients planned for FSS had positive lymph nodes; these women most often received chemoradiation therapy [6]. Correct prediction of LNM preoperatively could avoid radical surgery and decrease total morbidity. Pre-operative lymph node status could be investigated by CT scan, MRI or FDG-PET/CT. A recent meta-analysis of Woo et al. showed a sensitivity of 0.51 for CT, 0.57 for MRI and 0.58 for PET/CT, with specificities of, respectively, 0.87, 0.93 and 0.95 [78]. While PET/CT seems not to have added value above MRI, it may be helpful in the case of suspicious nodes on MRI. 

Only half of the lymph node metastases were diagnosed during surgery by FSA. Especially micro-metastases are missed during surgery. A positive lymph node results in upstaging to FIGO IIIC1 and therefore an indication for pelvic chemoradiotherapy. In 43 (41%) of patients with positive lymph nodes at FSA, the surgery changed into a radical hysterectomy. In 10 (10.5%) of women with a positive node after final histology, the (radical) hysterectomy was performed in a second surgical procedure. Leaving the uterus in place could reduce the risk of post-radiation toxicity such as acute bowel side effects and long-term risk of fistula since the uterus pushes the small bowel from the pelvic radiation field. Furthermore, a hysterectomy in the case of finding a positive node does not improve survival and is therefore not recommended for patients who require adjuvant radiation [79]. The presence of isolated tumor cells (ITC) does not cause upstaging to a higher stage [80]. In this study, none of the patients with ITC received any adjuvant treatment. So far, there does not seem to be any survival benefit from adjuvant chemoradiation. 

A small percentage (4.1%) of all women had positive cervical margins. Although there is no consensus about what should be considered a “free margin”, most studies suggest a minimum microscopic free margin of 5 mm [7]. The performance of FSA of the resection margin creates the possibility to remove extra tissue as long as the remaining cervical length allows this. Not all authors clearly reported how often this happened in their study population. We assume that when the extra removed tissue showed negative results on final histology, this was recorded as negative margins. Some authors performed a (radical) hysterectomy in the case of a positive margin and insufficient remaining cervix length in the same procedure, while others preferred to wait for final histology given the high impact to perform a hysterectomy in patients who wish to retain fertility [38].

In this review, only 0.3% of patients (11 patients) had parametrial involvement. This number is even lower in ECC with tumors <2 cm. Just recently, the results of the SHAPE trial have been published, a prospective randomized study in women with ECC (FIGO 2018 IA2 or IB1) between simple hysterectomy and radical hysterectomy with pelvic lymph node dissection. The SHAPE trial also found parametrial invasion in 0.3% of all patients. A radical hysterectomy resulted in more postoperative complications, without any survival benefit (3.1% recurrence versus 2.9% recurrence) [81]. From these results, we may infer that parametrectomy can probably be omitted in FSS for tumors < 2 cms.

In 209 (5.1%) patients, histology showed intermediate-risk features. Most authors did not use the Sedlis criteria to identify intermediate risk. Sedlis et al. showed an increase in recurrence from 2% to 31% in patients with stage IB cervical cancer treated with radical hysterectomy and pelvic lymphadenectomy and fulfilling the aforementioned Sedlis criteria. However, since Sedlis criteria did not differentiate between different histologic subtypes, Levinson et al. developed histology-specific nomograms to predict recurrence [9]. These nomograms suffer from too small patient numbers in the subgroups for adequate risk assessment. Nevertheless, if these criteria suggest a relatively low recurrence risk without adjuvant therapy, refraining from adjuvant therapy in order to preserve fertility might be a viable option in selected and well-informed patients [9].

So far, there is no debate on the safety of these minimally invasive procedures. Since the LACC trial, there is an ongoing debate on the oncological safety issues of minimally invasive radical hysterectomies [82]. Hopefully, the results of the RACC trial will help draw conclusions on oncological safety for robot-assisted procedures [83]. Nowadays, trachelectomy is also performed by minimally invasive surgery with laparoscopic or robotic assistance. Whether the concerns about oncological safety are applicable for FSS as well remains unclear. 

In terms of recommendations for the adjuvant treatment of patients with high- or intermediate-risk features after trachelectomy, no generally accepted guidelines exist for the adjuvant treatment of this patient group. Most experts will suggest adjuvant chemoradiotherapy in the event of high-risk features (nodal involvement, parametrial extension, involved margins). These recommendations are mostly inferred from studies with an unselected patient population who did not necessarily qualify for FSS [6]. One cannot immediately draw the same conclusion for patients qualified for FSS, as they are in general younger, usually have tumors < 2 cm and have undergone extensive imaging and/or sentinel node procedures to exclude nodal disease (resulting in limited/low-volume unexpected nodal involvement). 

Adjuvant pelvic radiation reduces the risk of recurrence as also shown in a meta-analysis by Rogers et al. [8,84]. Lee et al. treated patients with stage IB-IIA cervical cancer fulfilling Sedlis criteria with chemotherapy alone, resulting in 3-year DFS of 94.6% and 5-year OS of 90.6%. Instead of chemoradiation as adjuvant therapy in patients with childbearing wish and lymph node metastasis, chemotherapy could be considered as an alternative option since it seems to have less impact on fertility. For both platinum and taxanes, the exact impact on fertility is unclear; however, fertility will be preserved in most patients [85]. Platinum and taxanes are classified as intermediate-risk for gonadal toxicity. [86]. Matsuo et al. showed that in patients with pelvic and or para-aortic lymph node metastasis, adjuvant chemotherapy had similar recurrence rates (5-year rates 36.6% vs. 34.1%) and survival rates (24.7% vs. 21.8%) to those who received radiation-based therapy. However, recurrence patterns differed: chemotherapy was associated with a decreased risk of distant recurrence (19.2% vs. 24.6%) but an increased risk of local recurrence (23.9% vs. 14.3%) as compared to (chemo)radiation [87]. Okugawa et al. treated 23 patients with chemotherapy after FSS. Seven patients attempted to conceive; one of them delivered twice [54].

Furthermore, although the efficacy of (chemo)radiotherapy in preventing loco-regional recurrences in cervical cancer has been clearly established, this is less so for overall survival, especially in the subgroup of interest in this systemic review. This is probably due to the fact that local recurrences and, to a lesser extent, regional recurrences in unirradiated patients may be salvaged by radical chemoradiotherapy. This opens another option for well-informed patients with high-or intermediate-risk features: to watch and wait under close follow-up for 1–2 years and then try to conceive. This spares the patient the burden of very serious toxicity, at the cost of uncertainty and a possible extensive salvage treatment. If adjuvant radiotherapy is given, the extent of treatment (radiotherapy or chemoradiotherapy) also remains a matter of debate. The definition of the target volume is not obvious and may vary, depending on the indication, as well as the associated toxicity. Modern image-guided radiotherapy techniques allow for very precise target definition. Nonetheless, this will still affect fertility as the radiation tolerance of the ovaries is very low, and irradiation of only the cervix will likely affect the ability of the uterus to expand in the case of pregnancy and cause severe complications if a pregnancy might occur [88]. In conclusion, the optimal management of patients with either high- or intermediate-risk features remains unclear and should be based on a careful weighing of the available evidence in individual patients. Including these patients in a prospective cohort study will probably lead to more definitive evidence in the future.

### Limitations

In our study, limited data are available about recurrence and survival. Slama et al. retrospectively collected data of 733 patients from 44 institutions with FIGO stage IA1 with LVSI or ≥ IA2 with or without LVSI who underwent FSS. A total of 7% of those patients experienced recurrence after a median follow-up of 72 months, while 2.6% died of the disease. The risk of recurrence was higher in patients with tumors > 2 cm (19.4 vs. 5.7% HR 2.98) [89].

A lot of studies investigating FSS exclude patients with high- or intermediate-risk features from further analysis. Most of them shortly described the number of excluded patients in the methods section, and we collected all those data for this review. Some studies might not have mentioned the number of patients excluded, which could have led to selection bias. Most often, limited data about the patients with high- or intermediate-risk features were available. We were not able to analyze risk differences for high- or intermediate-risk features in patients with a certain grade, histology and stage. This is a limitation of the current study. In the future, we recommend authors to document the features of patients with high- or intermediate-risk features in future articles, in order to help to predict in which patients FSS is a suitable option. 

We included patients with all types of FSS except NACT, resulting in a wide overview of the subject. However, this might make it more difficult to apply the data to the individualized patient.

## 5. Conclusions

In more than 90% of women undergoing FSS, fertility could be preserved. Adjuvant chemotherapy after FSS could be an alternative treatment option from the aspects of effectiveness, safety and fertility preservation and could even increase this number. Adequate risk selection preoperatively with radiology or during surgery with FSA will help in clinical decision making. Parametrectomy may be omitted in the FSS of tumors less than 2 cm. 

## Figures and Tables

**Table 1:** Overview of articles with the amount of high- and intermediate-risk histologic features.

Author	Year	Cohort Size *n* (%)	Cervical Surgery	Lymph Node Surgery	Lymph Node Metastases *n* (%)	Positive Resection Margin *n* (%)	Parametrial Invasion *n* (%)	Unknown High Risk *n* (%)	Intermediate-Risk Factors *n* (%)
Ayhan [16]	2019	22	ART	LND	0	0	0	0	0
Bratila [17]	2016	36	RVT	SN + LND	5 (13.9%)	0	0	0	0
Breban-Kehl [18]	2022	75	RVT	SN + LND	0	0	1 (1.3%)	0	0
Burnett ^^^ [19]	2003	21	RVT	LND	2 (9.5%)	2 (9.5%)	0	0	0
Chen [20]	2008	16	LRT	LND	0	0	0	0	1 (6.3%)
Choi [21]	2014	22	cone	LND	1 (4.5%)	0	0	0	0
Cintra [22]	2016	17	ART	LND	1 (5.9%)	0	1 (5.9%)	0	0
Clark [23]	2016	28	RRT	LND	0	0	0	0	0
Dandan [24]	2014	39	ART	LND	3 (7.7%)	0	0	0	0
Demirkiran [25]	2018	14	SVT	SN ± LND	0	1 (7.1%)	0	0	0
Deng [26]	2017	49	ART	SN + LND	4 (8.2%)	0	0	0	16 (32.7%)
Doan [27]	2021	12	RT	LND	0	0	0	0	0
Ekdahl [28]	2022	166	ART	SN ± LND	8 (4.8%)	8 (4.8%)	0	0	1 (0.6%)
Fagotti [29]	2011	17	cone	LND	1 (5.9%)	5 (29.4%)	0	0	0
Fuij [30]	2012	156	ART	SN or LND	0	0	0	30 (19.2%)	0
Gent [31]	2014	28	ART	LND	0	0	0	0	0
Gil-Ibanez ^!^ [32]	2022	110	SVT/ART	LND	1 (0.9%)	1 (0.9%)	0	0	8 (7.3%)
Gil-Ibanez [33]	2020	38	SVT/RVT	LND	1 (2.6%)	1 (2.6%)	0	0	0
Guo [34]	2019	154	ART	SN or LND	0	4 (2.6%)	0	0	53 (34.4%)
Hauerberg ^!^ [35]	2015	122	RVT	SN	11 (9.0%)	0	0	0	0
Helpman ^$^ [36]	2011	160	RVT	LND	4 (2.5%)	3 (1.9%)	0	0	0
Hruda [37]	2021	91	cone	LND	2 (2.2%)	0	0	0	0
Ismiil ^$^ [38]	2009	132	RVT	LND	0	6 (4.5%)	0	0	0
Jeremic [39]	2009	12	ART	LND	1 (8.3%)	1 (8.3%)	0	0	0
Kanao [40]	2021	40	LRT	LND	1 (2.5%)	4 (10.0%)	0	0	0
Kathurusinghe [41]	2014	25	SVT	LND	2 (8.0%)	2 (8.0%)	0	0	0
Kim M [42]	2016	42	RVT	LND	0	2 (4.8%)	1 (2.4%)	0	0
Konishi [43]	2021	17	ART	LND	0	0	0	0	7 (41.2%)
Kucukmetin [44]	2014	27	LRT/ART	LND	4 (14.8%)	1 (3.7%)	0	0	0
Lanowska [45]	2011	225	RVT	LND	3 (1.3%)	3 (1.3%)	0	0	7 (3.1%)
Li [46]	2019	387	RVT	LND	40 (10.3%)	24 (6.2%)	0	0	71 (18.3%)
Lindsay [47]	2014	43	LLETZ	LND	1 (2.3%)	5 (11.6%)	0	0	0
Lu [48]	2013	25	LRT	LND	0	0	1 (4.0%)	0	0
Lucchini [49]	2021	32	cone	SN or LND	1 (3.1%)	0	0	0	0
Malmsten [50]	2018	39	RVT	LND	1 (2.6%)	10 (25.6%)	0	0	0
Marchiole [51]	2007	135	RVT	SN ± LND	9 (6.7%)	13 (9.6%)	0	0	0
Martinelli [52]	2021	39	cone	SN or LND	4 (10.3%)	17 (43.6%)	0	0	0
Matylevich ^<^ [13]	2021	77	SVT/ART	LND	7 (9.1%)	6 (7.8%)	0	0	0
Novikova [53]	2009	54	ART	SN or LND	6 (11.1%)	3 (5.6%)	0	0	0
Okugawa ^>^ [54]	2021	208	ART	LND	6 (2.9%)	1 (0.5%)	0	0	14 (6.7%)
Pareja [55]	2008	15	ART	LND	0	0	1 (6.7%)	0	0
Park [56]	2014	88	LRT	LND	0	0	0	9 (10.2%)	9 (10.2%)
Persson [57]	2012	25	RRT	LND	1 (4.0%)	3 (12.0%)	0	0	0
Plante ^>^ [14]	2011	136	RVT	SN + LND	15 (11.0%)	7 (5.1%)	0	0	0
Plante [58]	2017	35	SVT	SN ± LND	2 (5.7%)	0	0	0	0
Plante [59]	2020	50	SVT/cone	LND	4 (8.0%)	0	0	0	0
Poka [60]	2017	24	ART	SN + LND	4 (16.7%)	1 (4.2%)	0	0	4
Raju [61]	2012	66	SVT/RVT	SN + LND	2 (3.0%)	2 (3.0%)	0	0	0
Ramalingam [62]	2021	56	SVT/RVT	SN or LND	6 (10.7%)	5 (8.9%)	0	0	0
Rizzuto [63]	2019	19	RVT	LND	0	0	0	0	0
Saso [64]	2013	45	ART	LND	9 (20.0%)	3 (6.7%)	1 (2.2%)	0	0
Schlaerth [65]	2003	10	RVT	LND	0	2 (20.0%)	0	0	0
Shepherd [66]	2006	123	RVT	LND	7 (5.7%)	3 (2.4%)	2 (1.6%)	0	0
Sonoda [67]	2010	91	RT	LND	6 (6.6%)	1 (1.1%)	1 (1.1%)	0	1 (1.1%)
Svintsitsky [68]	2012	40	ART	LND	0	1 (2.5%)	0	0	0
Testa [69]	2013	30	ART	LND	1 (3.3%)	4 (13.3%)	0	0	0
Tomao [70]	2017	54	cone	LND	0	0	0	0	11 (20.4%)
Tsang [71]	2018	16	RT	SN ± LND	1 (6.3%)	0	0	0	0
Ungar [72]	2005	33	ART	LND	2 (6.1%)	1 (3.0%)	0	0	0
Vieira ^#^ [73]	2015	100	ART	LND	2 (2.0%)	8 (8.0%)	2 (2.0%)	0	0
Wang [74]	2019	88	RVT	LND	5 (5.7%)	0	0	0	0
Wu C [75]	2017	10	ART	LND	1 (10.0%)	1 (10.0%)	0	0	0
Yoo [76]	2016	12	LRT	LND	0	0	0	0	1 (8.3%)
Zusterzeel [77]	2016	132	RVT	LND	6 (4.5%)	3 (2.3%)	0	0	5 (3.8%)
Total		4118			212 (5.1%)	169 (4.1%)	11 (0.3%)	39 (0.9%)	209 (5.1%)

*n*: number; ART: abdominal radical trachelectomy; RVT: radical vaginal trachelectomy; LRT: laparoscopy-assisted radical trachelectomy; SVT: simple vaginal trachelectomy; RT: radical trachelectomy (not specified); LLETZ: large loop excision of the transformation zone; RRT = robot-assisted trachelectomy; LND = lymph node dissection; SN = sentinel node; ^^^ 1 patient with both positive margin and positive lymph node; ^#^ 1 patient with both positive resection margin and parametria; ^$^ the patients included in the study of Ismiil 2009 were also included in the study of Helpman 2011. For the total amount of patients, only the patients of Helpman were counted. The study of Ismiil was only used for the extra information given about the high-risk histologic features. ^<^ 6 patients with neo-adjuvant chemotherapy were excluded; ^>^ 4 patients with neo-adjuvant chemotherapy were excluded; ^!^ 1 patient with neo-adjuvant chemotherapy was excluded.

**Table 2:** Patients with high- and intermediate-risk histologic features during or after FSS.

Type of High- and Intermediate-Risk Histology *n* (%) ^1^	Type of Histology *n* (%) ^2^	Treatment after Histology Results *n* (%) ^2^	Fertility not Preserved *n* (%) ^2^
Positive lymph nodes *n* = 212 (5.1%)	FSA *n* = 105 (49.5%)	Hysterectomy ± adjuvant treatment *n* = 47 (44.8%)	*n* = 181 (85.4%)
	Final histology *n*= 95 (44.8%)	Hysterectomy ± adjuvant treatment *n* = 10 (10.5%) Extra lymph nodes *n* = 3 (3.2%) Radiotherapy *n* = 9 (9.5%) Chemoradiation *n* = 37 (38.9%) Chemotherapy *n* = 16 (16.8%) Unknown *n* = 7 (7.3%) None *n* = 14 ^#^ (14.7)	
	Unknown *n* = 12 (5.7%)	Hysterectomy *n* = 7 (58.3%) Radiotherapy *n* = 2 (25.0%) Unknown *n* = 3 (16.7%)	
Positive surgical margins *n*= 169 (4.1%)	FSA *n* = 88 (52.1%)	Hysterectomy ± adjuvant treatment *n* = 58 (65.9%) Unknown *n* = 30 (34.1%)	*n* = 144 (85.2%)
	Final histology *n* = 52 (30.7%)	Hysterectomy ± adjuvant treatment *n* = 19 (36.5%) Repeat LLETZ/conization *n* = 20 (385%) (Chemo)radiation *n* = 8 (15.4%) Chemotherapy *n* = 3 (5.6%) None *n* = 2 * (3.8%)	
	Unknown *n* = 29 (17.2%)	Hysterectomy *n* = 21 (72.4%) Unknown *n* = 8 (27.6%)	
Positive parametrial involvement *n* = 11 (0.3%)	FSA *n* = 1 (9.1%)	Radical hysterectomy *n* = 1 (9,1%)	*n* = 11 (100%)
	Final histology *n* = 10 (90.1%)	(Chemo)radiation *n* = 8 (72.7%) Unknown *n* = 2 (18.2%)	
Intermediate-risk factors *n* = 209 (5.1%)	FSA *n* = 2 (1.0%)	Hysterectomy *n* = 2 (1.0%)	*n* = 23 (11.0%)
	Final histology *n* = 207 (99.0%)	Hysterectomy ± adjuvant treatment *n* = 8 (3.8%) (Chemo)radiation *n* = 5 (2.4%) Chemotherapy *n* = 182 (87.1%) Adjuvant treatment *n* = 10 (4.8%) None *n* = 4 ^^^ (1.9%)	

FSS = fertility-sparing surgery; *n* = number; FSA = frozen section analysis; ^1^: percentage of total study population; ^2^: percentage of adverse histology; ^#^ 2 patients refused treatment; * 1 patient refused treatment; ^^^ 4 patients refused treatment.

**Table 3:** Occurrence of high- and intermediate-risk features and type of fertility-sparing surgery performed.

High- or Intermediate-Risk Features	Type of Fertility-Sparing Surgery Performed									
	RVT *n* (%)	ART *n* (%)	Cone *n* (%)	LRT *n* (%)	SVT *n* (%)	RT *n* (%)	RRT *n* (%)	LLETZ *n* (%)	SVT or ART *n* (%)	SVT or RVT *n* (%)
Lymph node metastases (*n* = 212)	103 (48.6%)	60 (28.3%)	16 (7.5%)	3 (1.4%)	10 (4.7%)	0	1 (0.5%)	5 (2.4%)	8 (3.8%)	6 (2.8%)
Positive resection margin (*n* = 169)	82 (48.5%)	33 (19.5%)	24 (14.2%)	5 (3.0%)	5 (3.0%)	5 (3.0%)	3 (1.8%)	0	6 (3.6%)	5 (3.0%)
Parametrial invasion (*n* = 11)	4 (36.4%)	5 (45.5%)	0	1 (9.1%)	0	1 (9.1%)	0	0	0	0
Intermediate-risk factors (*n* = 209)	83 (39.7%)	101 (48.3%)	11 (5.3%)	11 (5.3%)	2 (1.0%)	1 (0.5%)	0	0	0	0
Total (*n* = 638)	272 (42.6%)	199 (31.2%)	51 (8.0%)	20 (3.1%)	17 (2.7%)	7 (1.1%)	4 (0.6%)	5 (0.8%)	14 (2.2%)	11 (1.7%)

*n*: number; ART: abdominal radical trachelectomy; RVT: radical vaginal trachelectomy; LRT: laparoscopy-assisted radical trachelectomy; SVT: simple vaginal trachelectomy; RT: radical trachelectomy (not specified); LLETZ: large loop excision of the transformation zone; RRT = robot-assisted trachelectomy; node dissection (*n* = 8) or para-aortal lymph node dissection (*n* = 4). Besides extended surgery, 4 of them also received adjuvant chemotherapy or (chemo)radiation.

## Data Availability

Not applicable.

## Supplementary Materials

The following supporting information can be downloaded at https://www.mdpi.com/article/10.3390/cancers15153920/s1, Supplementary Material S1: Search strategy; Table S1: Risk of bias included in articles, Table S2: Intermediate-risk criteria.
