# Supplementary material for: High- or Intermediate-Risk Histologic Features in Patients with Clinical Early-Stage Cervical Cancer Planned for Fertility-Sparing Surgery: A Systematic Review
Source: Cancers (Basel). 2023 Aug 1;15(15):3920. doi: 10.3390/cancers15153920 (PMC10417237; doi:10.3390/cancers15153920)
Supplement: Supplementary file 1 [file cancers-15-03920-s001.zip › cancers-2513984-supplementary.pdf]

**Supplementary Material S1**

Part 1

("Uterine Cervical Neoplasms"[Mesh]

OR adeno squamous cell carcinoma*[Title/Abstract]

OR cervical adenocarcinoma*[Title/Abstract]

OR cervical cancer*[Title/Abstract]

OR cervical carcinoma*[Title/Abstract]

OR cervical neoplas*[Title/Abstract]

OR cervical squamous cell carcinoma*[Title/Abstract]

OR cervical tumo*[Title/Abstract]

OR adeno squamous cell carcinoma*[Title/Abstract]

OR cervix adenocarcinoma*[Title/Abstract]

OR cervix cancer*[Title/Abstract]

OR cervix carcinoma*[Title/Abstract]

OR cervix neoplas*[Title/Abstract]

OR cervix squamous cell carcinoma*[Title/Abstract]

OR cervix tumo*[Title/Abstract])

AND

Part 2

("Conization"[Mesh] OR "Trachelectomy"[Mesh] OR "Organ Sparing Treatments"[Mesh]

OR

(("surgery" [Subheading] OR "Gynecologic Surgical Procedures"[Mesh:NoExp]) AND ("Fertility Preservation"[Mesh] OR "Fertility"[Mesh] OR "Cervix Uteri"[Mesh]))

OR cervicectom*[Title/Abstract]

OR cone biops*[Title/Abstract]

OR Conisation*[Title/Abstract]

OR Conization*[Title/Abstract]

OR cone resection [Title/Abstract]

OR fertility preserv* [Title/Abstract]

OR fertility sparing [Title/Abstract]

OR Large loop excision of the transformation zone[Title/Abstract]

OR LLETZ[Title/Abstract]

OR trachelectom*[Title/Abstract])

AND

Part 3

("Lymph Nodes"[Mesh] OR "Lymph Node Excision"[Mesh] OR "Sentinel Lymph Node"[Mesh]

OR lymph node* [Title/Abstract]

OR Lymphadenectom*[Title/Abstract]

OR sentinel node* [Title/Abstract]

OR parametri*[Title/Abstract])

**Supplementary Table S1. Risk of bias of included articles**

| Author | Year | Confouding | Classification of interventions | Deviation from intended intervention | Missing data | Selection of patients |
|---|---|---|---|---|---|---|
| Ayhan | 2019 | + | + | + | + | ? |
| Bratila | 2016 | + | + | + | ? | + |
| Breban-Kehl | 2022 | ? | + | + | + | + |
| Burnett | 2003 | ? | + | + | ? | + |
| Chen | 2008 | + | + | + | + | + |
| Choi | 2014 | + | + | + | + | - |
| Cintra | 2016 | + | + | + | ? | + |
| Clark | 2016 | + | + | + | ? | - |
| Dandan | 2014 | + | + | + | - | - |
| Demirkiran | 2018 | ? | + | + | + | + |
| Deng | 2017 | + | + | + | ? | + |
| Doan | 2021 | + | + | + | + | - |
| Ekdahl | 2022 | + | + | + | + | + |
| Fagotti | 2011 | + | + | + | + | + |
| Fuij | 2012 | + | + | + | + | + |
| Gent | 2014 | + | + | + | + | + |
| Gil – Ibanez | 2022 | ? | + | + | + | + |
| Gil – Ibanez | 2020 | ? | + | + | + | + |
| Guo | 2019 | + | + | + | + | + |
| Hauerberg | 2015 | + | + | + | ? | + |
| Helpman | 2011 | + | + | + | + | ? |
| Hruda | 2021 | + | + | + | + | + |
| Ismiil | 2009 | + | + | + | ? | ? |
| Jeremic | 2009 | + | + | + | ? | ? |
| Kanao | 2021 | + | + | + | - | + |
| Kathurusinghe | 2014 | + | + | + | ? | ? |
| Kim M | 2016 | + | + | + | - | - |
| Konishi | 2021 | + | + | + | ? | + |
| Kucukmetin | 2014 | + | + | + | - | - |
| Lanowska | 2011 | + | + | + | - | - |
| Li | 2019 | + | + | + | ? | ? |
| Lindsay | 2014 | + | + | + | ? | ? |
| Lu | 2013 | + | + | + | + | ? |

| | | | | | | |
|---|---|---|---|---|---|---|
| Lucchini | 2021 | + | + | + | + | - |
| Malmsten | 2018 | + | + | + | ? | ? |
| Marchiole | 2007 | - | + | + | - | + |
| Martinelli | 2021 | ? | + | + | ? | + |
| Matylevich | 2021 | + | + | + | - | - |
| Novikova | 2009 | + | + | + | - | + |
| Okugawa | 2021 | - | + | + | ? | ? |
| Pareja | 2008 | ? | + | + | + | + |
| Park | 2014 | + | + | + | ? | ? |
| Persson | 2012 | + | + | + | ? | ? |
| Plante | 2011 | - | + | + | ? | + |
| Plante | 2017 | ? | + | + | + | + |
| Plante | 2020 | + | + | + | ? | ? |
| Poka | 2017 | ? | + | + | + | + |
| Raju | 2012 | ? | + | + | + | + |
| Ramalingam | 2021 | - | + | + | - | + |
| Rizzuto | 2019 | + | + | + | + | + |
| Saso | 2013 | + | + | + | ? | ? |
| Schlaerth | 2003 | + | + | + | ? | ? |
| Shepherd | 2006 | ? | + | + | + | + |
| Sonoda | 2010 | - | + | + | - | + |
| Svintsitsky | 2012 | + | + | + | ? | ? |
| Testa | 2013 | ? | + | + | ? | ? |
| Tomao | 2017 | + | + | + | ? | ? |
| Tsang | 2018 | - | + | + | - | + |
| Ungar | 2005 | + | + | + | ? | ? |
| Vieira | 2015 | ? | + | + | - | + |
| Wang | 2019 | + | + | + | - | + |
| Wu C | 2017 | + | + | + | - | + |
| Yoo | 2016 | + | + | + | - | - |
| Zusterzeel | 2016 | + | + | + | + | + |

n = number; green: low risk; yellow: medium risk; red: high risk

**Supplementary Table S2.** Intermediate-risk criteria

| Author | Year | Intermediate risk criteria | Number of patients / total study population | Fertility impairing treatment* | Chemotherapy |
|---|---|---|---|---|---|
| Chen | 2008 | LVSI | 1/16 | 0 | 1 |
| Deng | 2017 | Poor differentiation, LVSI, DSI | 16/49 | 0 | 16 |
| Ekdahl | 2022 | Sedlis | 1/166 | 1 | 0 |
| Gil-Ibanez | 2022 | unknown | 8/111 | 8 | 0 |
| Guo | 2019 | Adenocarcinoma, tumor size > 2 cm, LVSI, poor differentiation, DSI | 53/154 | 0 | 53 |
| Konishi | 2021 | Unknown | 7/17 | 0 | 7 |
| Lanowska | 2011 | LVSI, tumor size > 2 cm | 7/225 | 2 | 2 |
| Li X | 2019 | Unknown | 71/387 | 2 | 69 |
| Okugawa | 2021 | DSI, skip lesions in vagina, LVSI in cardinal ligament | 14/208 | 1 | 12 |
| Park | 2014 | LVSI | 9/88 | 0 | 9 |
| Poka | 2017 | Unknown | 4/24 | 4 | 0 |
| Sonoda | 2010 | DSI with LVSI | 1/91 | 1 | 0 |
| Tomao | 2017 | Unknown | 11/54 | 0 | 11 |
| Yoo | 2016 | Tumor size > 4 cm, LVSI | 1/12 | 1 | 0 |
| Zusterzeel | 2016 | Extensive LVSI, tumor size > 2 cm | 5/132 | 5 | 0 |

LVSI: lymph vascular space invasion; DSI: deep stromal invasion; Fertility impairing treatment: hysterectomy, radiotherapy or unknown fertility impairing treatment; * Fertility impairing treatment: hysterectomy, radiotherapy or unknown fertility impairing treatment.
